# Maternal Phylogeny of a Newly-Found Yak Population in China

**DOI:** 10.3390/ijms130911455

**Published:** 2012-09-12

**Authors:** Tserang Donko Mipam, Yongli Wen, Changxiu Fu, Shanrong Li, Hongwen Zhao, Yi Ai, Lu Li, Lei Zhang, Deqiang Zou

**Affiliations:** 1 Ecological Conservation and Animal Husbandry Research and Development Base of Qinghai-Tibetan Plateau, Southwest University for Nationalities, Chengdu 610041, China; E-Mail: tdmipam@163.com; 2 College of Life Science and Technology, Southwest University for Nationalities, Chengdu 610041, China; E-Mails: swun-zhw@163.com (H.Z.); neoacnew@gmail.com (Y.A.); lulu860620@yahoo.com.cn (L.L.); shitouji27@yahoo.com.cn (L.Z.); swunzdq@yahoo.com.cn (D.Z.); 3 Animal Husbandry Station of Sichuan Province, Chengdu 610041, China; E-Mail: fcxb1079@sina.com; 4 Animal Husbandry Bureau of Jinchuan County, Abazhou, Sichuan 624100, China; E-Mail: jcxmyj@163.com; 5 Sichuan Academy of Grassland Science, Chengdu 611731, China

**Keywords:** *Bos grunniens*, Jinchuan yak population, mtDNA control region, mitochondrial genome, genetic diversity, phylogeny

## Abstract

The Jinchuan yak is a new yak population identified in Sichuan, China. This population has a special anatomical characteristic: an additional pair of ribs compared with other yak breeds. The genetic structure of this population is unknown. In the present study, we investigated the maternal phylogeny of this special yak population using the mitochondrial DNA variation. A total of 23 Jinchuan yaks were sequenced for a 823-bp fragment of D-loop control region and three individuals were sequenced for the whole mtDNA genome with a length of 16,371-bp. To compare with the data from other yaks, we extracted sequence data from Genebank, including D-loop of 398 yaks (from 12 breeds) and 55 wild yaks, and whole mitochondrial genomes of 53 yaks (from 12 breeds) and 21 wild yaks. A total of 127 haplotypes were defined, based on the D-loop data. Thirteen haplotypes were defined from 23 mtDNA D-loop sequences of Jinchuan yaks, six of which were shared only by Jinchuan, and one was shared by Jinchuan and wild yaks. The Jinquan yaks were found to carry clades A and B from lineage I and clade C of lineage II, respectively. It was also suggested that the Jinchuan population has no distinct different phylogenetic relationship in maternal inheritance with other breeds of yak. The highly haplotype diversity of the Pali breed, Jinchuan population, Maiwa breed and Jiulong breed suggested that the yak was first domesticated from wild yaks in the middle Himalayan region and the northern Hengduan Mountains. The special anatomic characteristic that we found in the Jinchuan population needs further studies based on nuclear data.

## 1. Introduction

**Figure 1:**
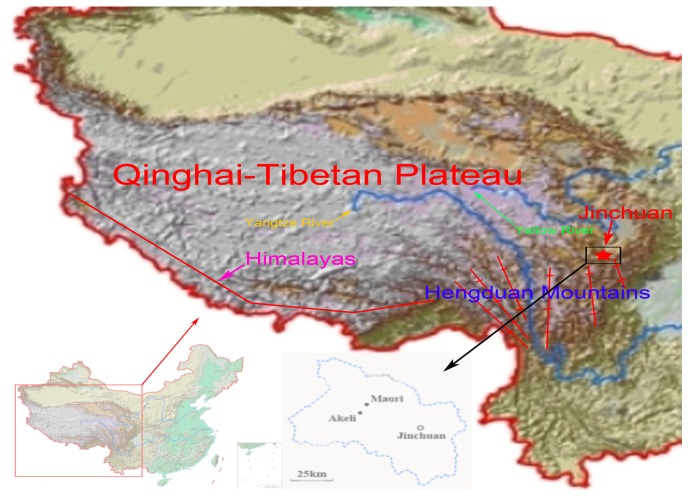
The distribution of Jinchuan yaks in Qinghai-Tibetan Plateau.

**Figure 2:**
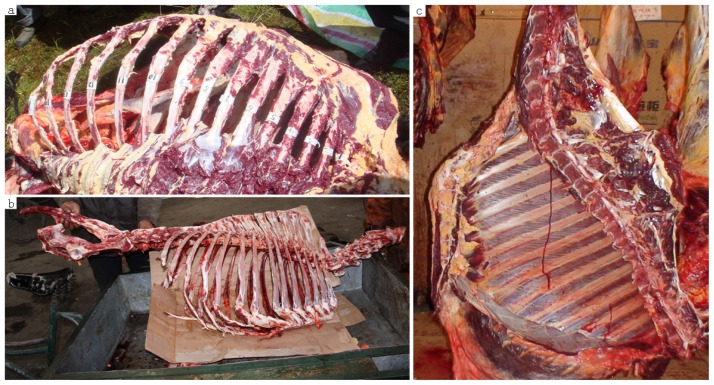
The skeleton of Jinchuan (**a**), Maiwa (**b**) and Jiulong (**c**) yak. We can find 15 ribs of one side in **a**, 14 ribs of one side in **b** and **c**.

The domestic yak (*Bos grunniens*) is the symbolic animal living in alpine climates (between 2000 to 5000 m) on the Qinghai-Tibetan Plateau (QTP) and adjacent highlands, such as Mongolia, Nepal, Bhutan, India, Pakistan, Afghanistan and countries of the Commonwealth of Independent States [1,2]. Few other domesticated animals could survive in such an environment. Over 95% of the yaks all over the world live in the QTP [2–4]. The yaks provide meat and milk, transportation, dung for fuel and hides for shelter. There are two major yak breeds that live in the Sichuan province of China: the valley-type Jiulong breed and the plateau grassland type Maiwa breed [2,3]. Recently, the Jinchuan yaks were observed in Maori and Akeli Village of Jinchuan County in Sichuan province. This region looks like an islet surrounded by high mountains and deep valleys (Figure 1). The geographic profile is latitudinal (101°40′E to 101°41′E) and longitudinal (31°32′N to 31°34′N). Compared with the valley type Jiulong breed and the plateau grassland type Maiwa breed, the Jinchuan yak has several special characteristics. First, recent investigation showed that 52% of Jinchuan yak individuals have 15 pairs of ribs while all other yaks have 14 pairs (Figure 2). Second, the reproductive rate of the Jinchuan yak (95%) is significantly higher than that of other breeds (70%~75%). Third, the Jinchuan yaks provide a better quality of milk and a higher yield of meat, and have a more powerful resistance to natural pressures. However, little investigation has been undertaken on the genetics of this population.

Mitochondrial DNA (mtDNA) is maternally inherited and has been widely used in phylogenetic studies. In particular, the control region D-loop has the highest genetic variation on mtDNA and has been used to investigate the maternal phylogenetic relationships among mammals [5–10]. Tu *et al*. analyzed D-loop region in 21 yaks using RFLP (Restriction Fragment Length Polymorphisms) and identified four haplotypes [11]. Lai *et al*. found that the domestic yaks converged into two mainly phylogeny groups, based on the DNA sequences of D-loop region and cytochrome b [12]. It was also found that there was no geographical differentiation between these two phylogeny groups. Similar results were observed in the other studies, based on mtDNA [13,14], blood protein electrophoresis [15], or nuclear microsatellite alleles [16]. Whole mtDNA sequencing has been shown to be more powerful in mammal phylogenetic studies than single gene, or region or nuclear genes [3,17–26].

In the present study, we will investigate the genetic diversity of a special yak population, Jinchuan yak, and the phylogenetic relationship with other yak breeds using both control region and whole mtDNA sequencing data.

## 2. Results and Discussion

### 2.1. Results

A fragment of 823 bp of the mtDNA control region was sequenced in 23 Jinchuan yaks. All of these sequences have been deposited in GenBank with the Accession numbers from JQ811490 to JQ811512. The whole mtDNA was sequenced in three Jinchuan yaks via six fragments, 2688 bp, 4190 bp, 3942 bp, 2798 bp, 3293 bp, and 3523 bp, respectively. The three assembled complete mtDNA sequences were deposited in GenBank with the accession numbers from JQ846020 to JQ846022.

#### 2.1.1. Nucleotide and Haplotype Diversity

**Figure 3:**
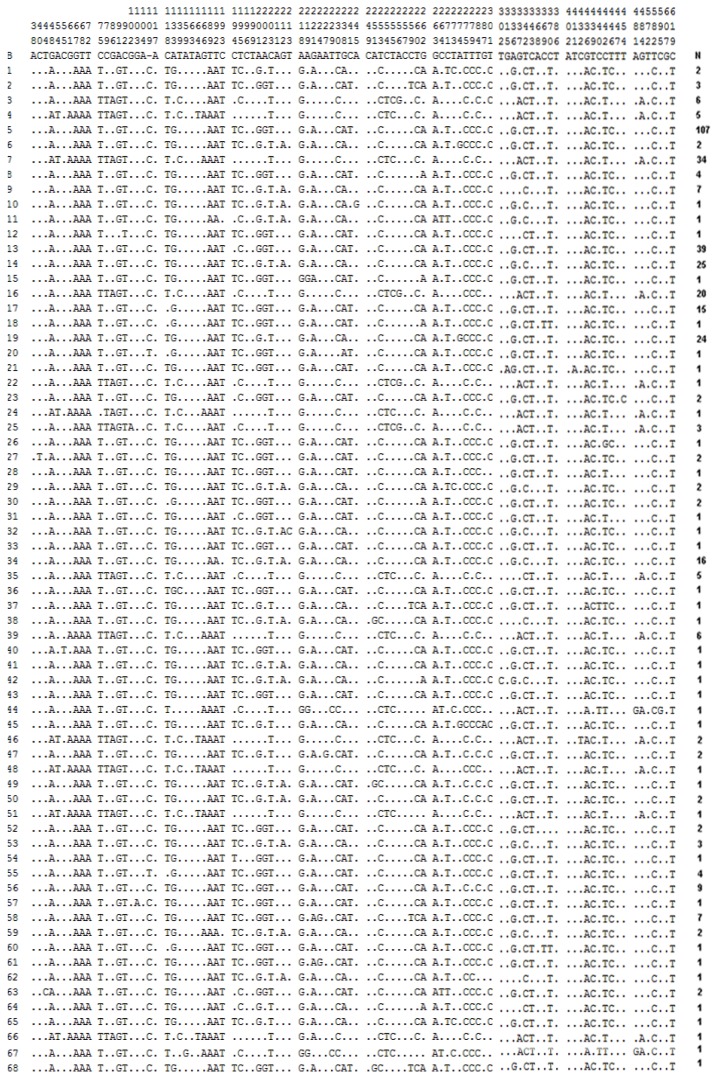

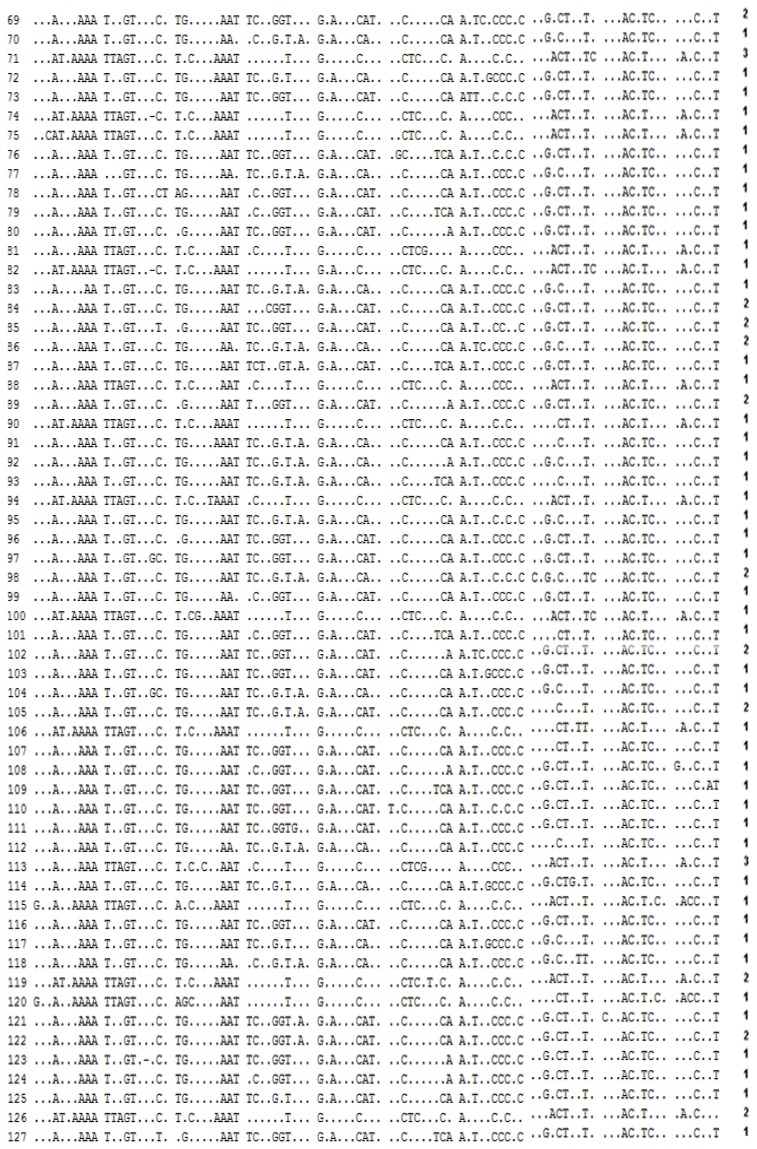
Nucleotide variations of 127 haplotypes in 476 *Bos grunniens* individuals based on mtDNA control regions. N in the right column listed the number of individuals sharing the same haplotype. Mutations are scored relative to the reference sequence of *Bison bison* (abbreviated as 1, Accession No. U12936). Numbers at the top of the figure indicate the nucleotide sequence position. Dots denote identity with the reference sequence.

For the mtDNA control region, we also downloaded data of 398 yaks (from 12 breeds) and 55 wild yaks from Genebank. Together with the control region sequence data from the 23 Jinchuan yak individuals, a total of 476 sequences were obtained with 638 bp available on each sequence. Substitution analyses of these 476 sequences revealed 97 variable sites (Figure 3) and the global nucleotide diversity (Pi) was 0.01614 ± 0.00069. In total, 127 haplotypes were identified with a haplotype diversity (Hd) of 0.9280 ± 0.0083 (Table 1). In the 23 Jinchuan yak individuals, 33 nucleotide variants were observed and 13 haplotypes were identified. Thirty-two haplotypes were identified in the 55 wild individuals and only one haplotype was shared between Jinchuan and wild yaks. The Jinchuan yaks showed higher genetic diversity (Pi = 0.02035 ± 0.00225, Hd = 0.925 ± 0.035) than most other breeds including Tianzhu, Gannan, Datong, Huanhu, Plateau, Jali, Sibu, Pali, Maiwa, Jiulong and Bazhou, but a somewhat smaller genetic diversity than the Zhongdian yak (Pi = 0.02210 ± 0.00385, Hd = 0.917 ± 0.092) (Table 1).

Each of the mitochondrial genomic sequence was 16371 bp in length. For the 77 individuals (Table 1), 378 variable sites were identified with a nucleotide diversity (Pi) of 0.00319 ± 0.00027, from which 68 haplotypes were defined with a haplotype diversity (Hd) of 0.9945 ± 0.005.

The coding regions of complete mitochondrial sequences were also used for further analysis, and all the 13 protein-coding genes were employed except the ND6 gene because of the significant codon usage bias [14]. The initiation and termination codons and overlapping regions between ATP6 and ATP8, ND4 and ND4L, and ND5 and ND6 were also excluded. The coding region of each mitochondrial genomic sequence was 10,710 bp in length and contained 3570 codons. In this data set, 228 nucleotide substitutions were identified, with a nucleotide diversity (Pi) of 0.00270 ± 0.00025 and 52 haplotypes were defined with a haplotype diversity (Hd) of 0.9450 ± 0.021, and three of them contain Jinchuan yak individuals.

#### 2.2.2. Phylogenetic Analysis

##### 2.2.2.1. Phylogeny Inferred from mtDNA Control Region

**Figure 4:**
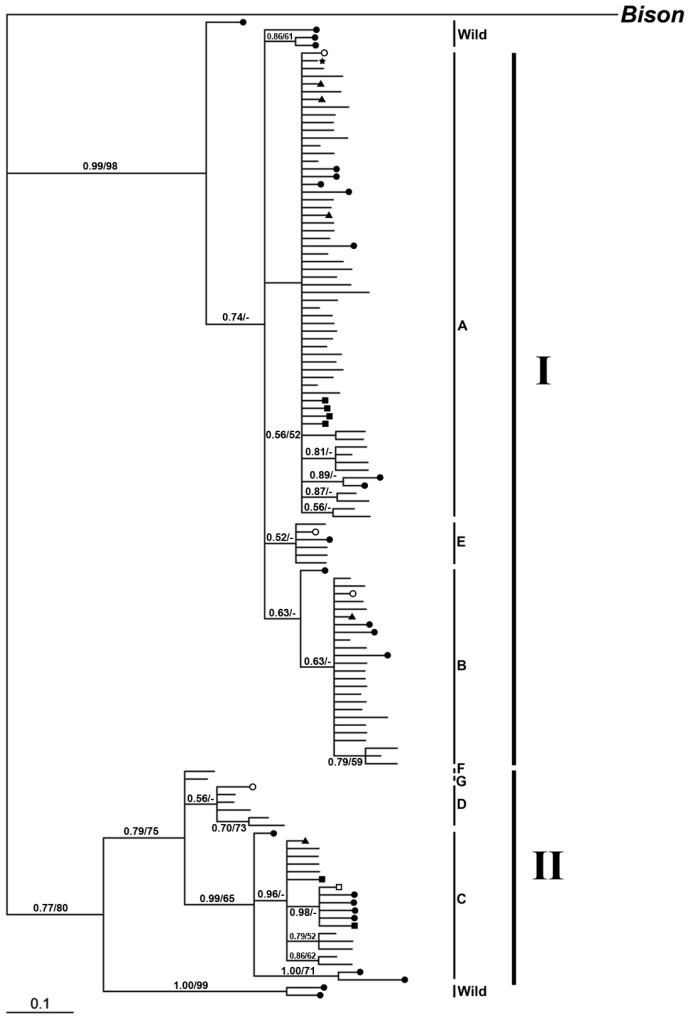
Phylogenetic tree of Jinchuan population and other 12 domestic yak breeds, together with a wild population basis of mtDNA control region constructed by neighbor-joining and Bayesian analysis, rooted by a bison sequence. ● indicates the haplotypes only found in the wild population; ○ indicates the haplotype shared by wild and yaks excluding the Jinchuan population; ■ indicates the haplotypes only found in the Jinchuan population; □ indicate the haplotypes shared by the Jinchuan population and wild yaks; ▼ indicates the haplotypes shared by the Jinchuan population and other 12 breeds; ★ indicate the haplotypes shared by all yaks, including wild, the Jinchuan population and other 12 breeds of yaks. The numbers at the nodes before the split means Bayesian posterior probabilities and after the sprit indicate the bootstrap values for 1000 Kimura two-parameter distance replications.

A neighbor-joining (NJ) tree based on the control region sequences showed that all the haplotypes fell into two distinct lineages (Figure 4). Lineage I diverged into clade A, B, E and Lineage II into clade C, D, F, G. Both lineages included the haplotypes of wild yak. The yak haplotypes were distributed randomly in the six clades (Figure 4). The Jinchuan yaks were found in the clade of A, B and C. Six Jinchuan haplotypes belonged to the clades of A and C. One haplotype in the clade of A was shared between Jinchuan, wild and domestic yaks. Five haplotypes (three in clade A, one in clade B and the other one in clade C) were shared between Jinchuan and other breeds of yak in the clade of A, B and C. Another haplotype in the clade C was only shared between Jinchuan and wild yaks (Table 2 and Figure 4).

For the mtDNA control region, the best model computed for Bayesian analysis was HKY + I + G, with a proportion of invariable sites of 0.7105 and a gamma distribution shape parameter of 0.7505. Bayesian analysis essentially produced the same topology as the previous analyses (NJ).

**Figure 5:**
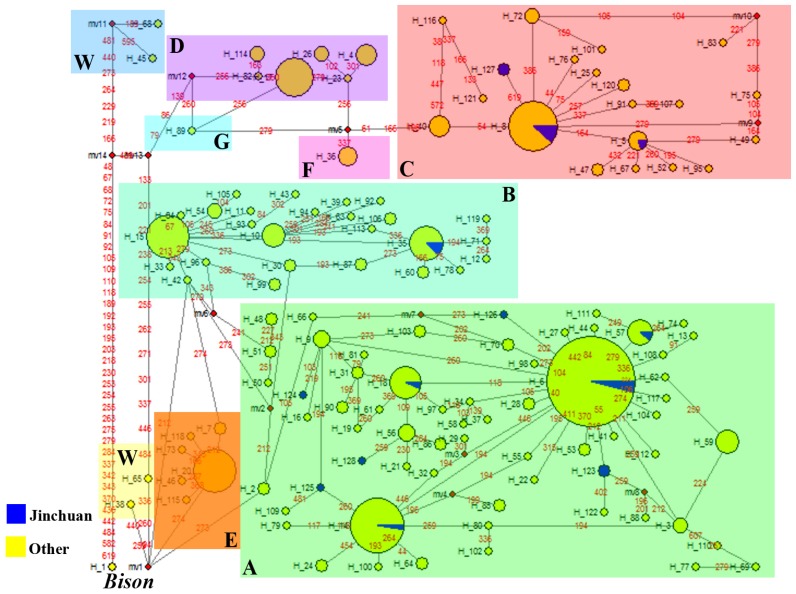
Median-joining network of haplotypes of the mtDNA control region for yaks and bison. The larger circles represent haplotypes and their sizes proportional to the frequency of individuals comprised. Mutational sites are shown on lines and median-joining sites are indicated by smaller circles. Shaded rectangle-shaped areas indicate phylogenetic lineages.

Median-joining network for the 128 haplotypes (including one bison haplotype) of mtDNA control region also revealed that all haplotypes of yaks converged into two genetic lineages (I and II), which diverged into seven clades (A–G) (Figure 5).

##### 2.2.2.2. Phylogeny Inferred from Coding Regions of Mitochondrial Genomic Sequences

**Figure 6:**
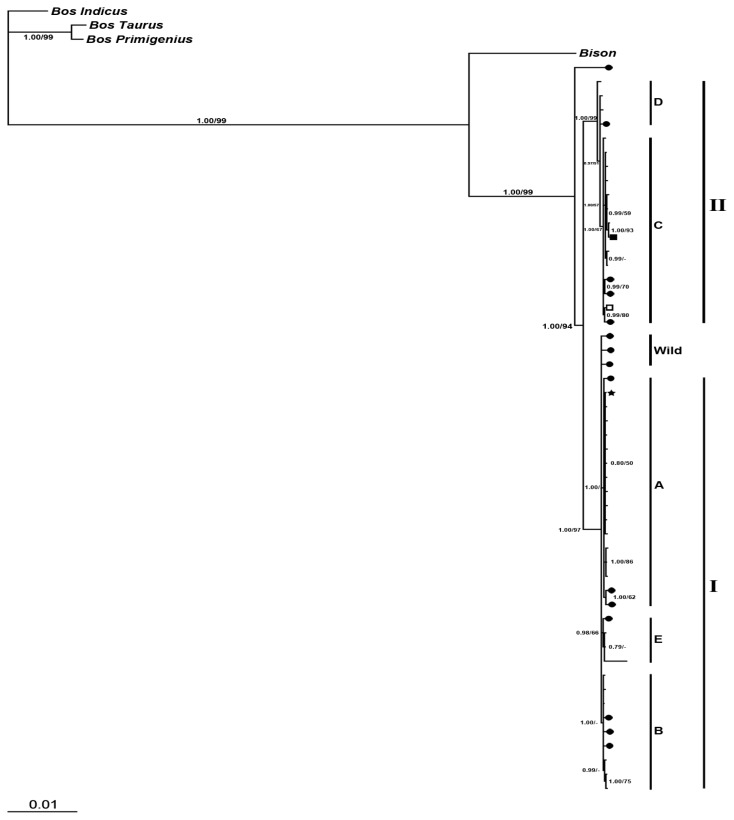
Phylogenetic tree of Jinchuan population and other 12 domestic yak breeds together with wild population and three sequences from bison, *Bos taurue* and *Bos primigenous* respectively, which is the basis of the coding region of complete mitochondrial sequences constructed by neighbor-joining and Bayesian analysis, rooted by a *Bos indicus* sequence. ● indicate the haplotypes only found in wild population; ■ indicate the haplotypes only found in Jinchuan population; □ indicate the haplotypes shared by Jinchuan population and wild yaks; ★ indicate the haplotypes shared by all yaks including wild and Jinchuan population and 12 other breeds of yak. The numbers at the nodes before the split means Bayesian posterior probabilities and after the sprit indicate the bootstrap values for 1000 Kimura two-parameter distance replications.

A neighbor-joining tree was constructed from the coding regions of mitochondrial genomic sequences for all domestic breeds and wild yaks, together with *Bos taurus* and *Bos primigenius*, with *Bos indicus* as the outgroup. All sequences again converged into two distinct lineages (Figure 6), comprising five clades (A–E). The Jinchuan yak haplotypes are in clades A and C, and the Jinchuan-specific haplotype belongs to clade C. One Jinchuan haplotype was located in clade A. This haplotype was also observed in other yaks, including 1 Pali, 1 Huanhu, 2 Tianzhu, 3 Ganlan, 1 Plateau, 1 Jiali, 2 Datong, 2 Maiwa and 1 Jiulong individual, together with 1 wild yak. Another haplotype in clade C was shared only by 1 Jinchuan yak and 1 wild yak. In this study, the Bison was found to be more closely related to yak than to other Bovid species. In addition, *Bos taurus* and *Bos primigenius* were clustered into the same clade and indicated the most closely related phylogeny (Figure 6).

Modeltest 3.7 showed that HKY+G was the best model computed for Bayesian analysis, with a proportion of invariable sites of 0 and a gamma distribution shape parameter of 0.0860. Again, Bayesian analysis produced approximately the same topology as the previous analyses (NJ).

### 2.2. Discussion

We presented the nucleotide and haplotype diversity of the yaks by breed instead of administrative division [13,14], which may not represent the real environment conditions of each breed. The highest haplotype diversity was observed in Datong yak, which is a hybrid of wild and domestic yaks. Among those indigenous breeds, Pali, Jinchuan, Maiwa and Jiulong exhibited the highest haplotype diversity. On the other hand, Zhongdian showed the highest nucleotide diversity followed by Jinchuan, Sibu, Gannan and Maiwa. Both nucleotide and haplotype diversity indicated that the Pali, Jinchuan, Maiwa and Jiulong showed more extensive genetic diversity than any other yak in China. This is because the yaks with high haplotype diversity were distributed in the middle Himalayan region, and north of the Hengduan Mountains. Guo *et al*. and Savolainen *et al*. showed that haplotype and nucleotide diversity in the populations near to a center of initial origin was higher than in the populations derived through subsequent migration colonization [13,27]. Based on the results in the present study, the yak was probably first domesticated at these areas from wild yaks, and then extended north and west until the yaks distributed throughout the QTP of western China. This does not agree with the prediction by Wang *et al*. [14], that yaks were first domesticated in Qinghai and Tibet province in China.

Special anatomic characteristics were found in the Jinchuan population. Unfortunately, the analysis of the phylogeny did not show distinct differences between Jinchuan and other breeds and wild yaks as well. Nevertheless, six specific mtDNA haplotypes were observed among eight individuals. In addition, one haplotype was shared by one Jinchuan yak individual and four wild yak individuals. Thus, it could be hypothesized that the ancestor of the Jinchuan population in wild yaks before domestication was a special group with similar characteristics to the Jinchuan population at that time, and then some of this special group were caught and domesticated by the hunters. This hypothesis can be supported not only by the mitochondrial control region sequences, but also by the mtDNA coding region sequence.

Here, we also employed coding regions of mtDNA from bison, *Bos taurue* and *Bos primigenous* respectively to construct the phylogenetic tree. Tree topologies showed that yak have a closer relationship with bison and a more distant relationship with two other bovid species: *Bos taurus* and *Bos primigenius*. Therefore, we propose to include domestic and wild yak in a separate subgenus *Poephagus* instead of the genus *Bos* [2,12].

## 3. Experimental Section

### 3.1. Sample Collection and DNA Extraction

Blood samples of 23 yaks (Jinchuan yak population) were collected from Reta village of Jinchuan county, Sichuan province and taken back to the lab, and maintained at −70 °C. Genomic DNA was extracted from blood using the standard extraction kit according to the manual of the TIANamp Blood DNA Kit (Tiangen, Beijing, China).

### 3.2. PCR Amplification and Sequencing

The fragment of the mtDNA control region of each sample was amplified by PCR using the primers we designed from the mtDNA genome of *Bos grunniens* (GenBank Accession No. GQ464314): F (5′-GTAAAGAGCCTCACCAGTAT-3′) and R (5′-GTCGGGAGACTCATCTAGGC-3′). PCR amplifications were carried out in a 50 μL reaction mixture containing 200 ng of genomic DNA, each primer (2 μL of a 10 μM solution), dNTPs (4 μL of a 2.5 mM solution), 5 μL of 10× Ex Taq buffer and 0.4 μL of 5 U μL^−1^ Ex Taq DNA polymerase (TaKaRa, Dalian, China). The PCR program, run in a Mastercycler pro, Thermal Cyclers (Eppendorf, Hamburg, Germany), consisted of an initial denaturing at 95 °C for 4min, followed by 35 cycles of 55 s at 95 °C, 55 s at 50 °C, 55 s at 72 °C, and a final 5 min extension at 72 °C. For complete mitochondrial sequencing, mitochondrial DNA fragments were amplified using a long and accurate-polymerase chain reaction (LA-PCR) kit (Takara, Dalian, China) and it was amplified using the primers and methods described by Wang *et al*. (2010) [14]. PCR products were purified using an AxyPrepTM DNA Gel Extraction Kit (Axygen, Union City, CA, USA) and sequenced on an ABI 3730xl automated sequencer at Invitrogen, Shanghai. Both strands of the PCR products of the fragment of the mtDNA control region were completely sequenced. The primers were used for sequencing complete mitochondrial data as shown in Table 3. All results were assembled by the program DNAman and manually to a continuous sequence.

### 3.3. Nucleotide and Haplotype Diversity

All the sequences of mtDNA control region and complete mitochondrial data from the Jinchuan population and GenBank (Accession No. of 453 mtDNA control region sequences were FJ548840 to FJ548845, GQ464116 to GQ464245, DQ856602 to DQ856604, DQ856594 to DQ856600, DQ138998 to DQ139260, DQ007210 to DQ007224, AY521137 to AY521161, AY722118, AY749414, AY374125, AF083355 and 74 complete mitochondrial sequences were GQ464246 to GQ464314, NC_006380, EF494179, EF494178, EF494177, AY684273.) were aligned using the ClustalX program [28]. Nucleotide variable sites of nucleotide substitution were explored by MEGA Version 4.0 [29]. Nucleotide diversity (Pi) and haplotype diversity (Hd) for all yaks were performed in DnaSP Version 5.0 [30].

### 3.4. Phylogenetic Analysis

Neighbor-joining (NJ) trees [31,32] of all the haplotypes for mtDNA control region and complete mitochondrial were constructed based on a Kimura 2-parameter model in MEGA Version 4.0, with the reliability of the tree topology assessed by 1000 replications. Bootstrap consensus tree was selected with hidden values lower than 50%.

The Bayesian analyses were also carried out for phylogenetic analyses. The program Modeltest [33] was utilized to find the model of sequence evolution that best fit data set by the hierarchical likelihood ratio test (hRLTs), though the Bayesian information criterion (BIC) proved to be best for model selection [34], in order to compare the result of phylogenetic inference with Wang *et al*. (2010), we still use hRLTs for model selection [14]. The best suggested model was subsequently used for Bayesian analysis in MrBayes 3.1 [35]. The tree-space was explored by four chains over 10 million generations with a sampling frequency of every 1000th generation. Independent runs were considered converged with a standard deviation of split frequencies of lower than 0.01 and potential scale reduction factor (PSRF) of 1.0 for all parameters. We obtained 10,000 trees, fixing the burn-in value at 25%. The first 2.5 million generations of both runs were discarded as burn in, and the remaining 7.5 million generations (7500 trees) were summarized in a 50% majority rule consensus tree.

Median-joining network [36] was constructed using Network 4.6 (www.fluxus-engineering.com) to investigate the possible relationships among haplotypes of mtDNA control region.

## 4. Conclusions

In summary, we observed six haplotypes in the Jinchuan population, but not in any of the other yak breeds or wild yaks. However, phylogenetic analyses did not distinguish Jinchuan from other breeds. The highly haplotype and nucleotide diversity of yaks in the Jinchuan population and breeds of Pali, Maiwa and Jiulong suggest that yak were perhaps first domesticated from wild yaks in the middle Himalayan region and north of the Hengduan Mountains. The special anatomic characteristic that we found in the Jinchuan population needs to be further studied, based on nuclear data.

## Figures and Tables

**Table 1:** Genetic diversity of *Bos grunniens* by Jinchuan population and other 12 domestic breeds together with wild population.

Breed or population	*N*	*H*	Hd ± SD	Pi ± SD	*A*	*B*	Nc
Jinchuan	23	13	0.925 ± 0.035	0.02035 ± 0.00225			3
Tianzhu	21	9	0.829 ± 0.066	0.00997 ± 0.00281	7	0.333333	4
Gannan	7	5	0.905 ± 0.103	0.01857 ± 0.00529	3	0.428571	7
Datong	31	19	0.942 ± 0.030	0.01582 ± 0.00271	13	0.419355	2
Huanhu	61	18	0.800 ± 0.048	0.01218 ± 0.00206	9	0.147541	5
Plateau	88	30	0.911 ± 0.017	0.01393 ± 0.00167	48	0.545455	14
Jiali	42	21	0.912 ± 0.031	0.01593 ± 0.00222	21	0.500000	1
Sibu	12	8	0.909 ± 0.065	0.01923 ± 0.00288	4	0.333333	2
Pali	40	19	0.933 ± 0.021	0.01733 ± 0.00220	19	0.475000	4
Maiwa	23	14	0.925 ± 0.041	0.01856 ± 0.00280	12	0.521739	6
Jiulong	12	8	0.924 ± 0.057	0.01403 ± 0.00477	5	0.416667	2
Bazhou	33	7	0.822 ± 0.035	0.00579 ± 0.00039	21	0.636364	0
Zhongdian	9	7	0.917 ± 0.092	0.02210 ± 0.00385	4	0.444444	1
wild	55	30	0.961 ± 0.012	0.02207 ± 0.00111	9	0.163636	23
unclear	6						3
Gansu unclear	8						
Sichuan unclear	5						
Total	476						77

Note: *N* stands for the number of individuals; *H* stands for the number of haplotypes; Hd indicates the haplotype diversity (Mean ± SD) and Pi means the nucleotide diversity (Mean ± SD). *A* stands for the number of yaks that shared the same haplotype with the Jinchuan population in each breed, and *B* shows the percentage of A in each breed. Nc indicates the number of individuals in each breed used for complete mitochondrial genomic sequencing.

**Table 2:** The number of individuals in each breed and wild population which shared the haplotype with the Jinchuan population.

Lineages	Clades	The haplotypes shared by Jinchuan and other yaks	The number of individuals from different populations harbored in the haplotypes
Lineage I	A	H6	4JC, 1ZD, 7PL, 2HH, 7TZ, 1GL, 19P, 7BZ, 3SB, 11JL, 7DT, 3MW, 3JL, 6W, 2SU
		H14, H18, H57	3JC, 6PL, 5HH, 19P, 4BZ, 7JL, 4DT, 9MW, 2JL, 4GU
	B	H35	2JC, 1GL, 1P, 10BZ, 1JL
Lineage II	C	H8	5JC, 3ZD, 6PL, 2HH, 1GL, 9P, 1SB, 2JL, 2DT, 2MW, 1GU
		H5	1JC, 4W

Note: JC-Jinchuan, ZD-Zhongdian, PL-Pali, HH-Huanhu, TZ-Tianzhu, GL-Ganlan, P- Plateau, BZ-Bazhou, SB-Sibu, JL-Jiali, DT-Datong, MW-Maiwa, JL-Jiulong, W-Wild, SU-Sichuan unclear, GU-Gansu unclear.

**Table 3:** Primers used for sequencing the complete mitochondrial genome.

PCR primer set	Primer name	Sequence(5′–3′)
1st pair	F1	AAATGACGAAAGTGACCCTA
	R1	TAGGGCTCCGATTAGTGCGT
	F1F06	AGAAAGTACCGCAAGGGA
	R1R06	ATGAGCGATAGAGTGATTTGAC
2nd pair	F2	CCTACGTGATCTGAGTTCAG
	R2	TGAGCCCATTGATGAGACAG
	F2F08	CAAACATGGCTAATCCTCC
	R2R08	GGAGTAATAGTACGGCGGTG
	F2F09	AACCCACGAGCTACAGAA
	F2F10	CATCCTAATCCTCGCCACT
3rd pair	F3	TCTACTATTTGGAGCCTGGG
	R3	ACGAAATGTCAGTATCAGGC
	F3F30	GAGCTATAATGTCAATCGGA
	R3R30	TATTAAGAGGGCGGATAGAG
	F3F31	TTTCAAGCCAACACCATAAC
4th pair	F4	AAGCCCTTGACCCCTTACAG
	R4	TCGTGTAAAGGAAGGTGAGA
	F4F02	TTTGACTTTTCCTCTATGTTTC
	R4R02	GAGTATTAGGAAGTTTAGGGATC
5th pair	F5	CAAACGGACCTAAAATCACT
	R5	GTGTATTGCTAGGAATAGGC
	F5F04	AATCTTCCAACTACCCGCTCTA
	R5R04	AGTTATTGTAACTGGGTGGTCT
6th pair	F6	AGAAAACCCTACGAAACCAA
	R6	CTTTCATCGTTCCCTTGCGG
	F6F06	CAGGCTCCAACAATCCAA
	R6R06	ATCGGCTGTTGTAGGGTC
	F6F08	GGGGATGCTTGGACTCAG
	F6F07	TGTAAAGAGCCTCACCAGTA
